# Enhanced Capacity Retention of Li_3_V_2_(PO_4_)_3_-Cathode-Based Lithium Metal Battery Using SiO_2_-Scaffold-Confined Ionic Liquid as Hybrid Solid-State Electrolyte

**DOI:** 10.3390/molecules28134896

**Published:** 2023-06-21

**Authors:** Shihao Peng, Jiakun Luo, Wenwen Liu, Xiaolong He, Fang Xie

**Affiliations:** College of Physical Science and Engineering Technology, Yichun University, Yichun 336000, China; pengshihao2003@163.com (S.P.); ssccal@163.com (J.L.); h2798678215@163.com (X.H.)

**Keywords:** Li_3_V_2_(PO_4_)_3_, lithium metal battery, solid-state electrolyte, cycling performance

## Abstract

Li_3_V_2_(PO_4_)_3_ (LVP) is one of the candidates for high-energy-density cathode materials matching lithium metal batteries due to its high operating voltage and theoretical capacity. However, the inevitable side reactions of LVP with a traditional liquid-state electrolyte under high voltage, as well as the uncontrollable growth of lithium dendrites, worsen the cycling performance. Herein, a hybrid solid-state electrolyte is prepared by the confinement of a lithium-containing ionic liquid with a mesoporous SiO_2_ scaffold, and used for a LVP-cathode-based lithium metal battery. The solid-state electrolyte not only exhibits a high ionic conductivity of 3.14 × 10^−4^ S cm^−1^ at 30 °C and a wide electrochemical window of about 5 V, but also has good compatibility with the LVP cathode material. Moreover, the cell paired with a solid-state electrolyte exhibits good reversibility and can realize a stable operation at a voltage of up to 4.8 V, and the discharge capacity is well-maintained after 100 cycles, which demonstrates excellent capacity retention. As a contrast, the cell paired with a conventional liquid-state electrolyte shows only an 87.6% discharge capacity retention after 100 cycles. In addition, the effectiveness of a hybrid solid-state electrolyte in suppressing dendritic lithium is demonstrated. The work presents a possible choice for the use of a hybrid solid-state electrolyte compatible with high-performance cathode materials in lithium metal batteries.

## 1. Introduction

The development of lithium batteries with a high energy density, safety, and cycling stability is crucial for promoting sustainable energy development [1,2]. For the anode, lithium metal is an ideal material due to its high theoretical capacity of 3860 mA h g^−1^ and low electrochemical potential of −3.04 V vs. standard hydrogen electrode [3,4]. However, when the lithium anode is assembled into batteries with a traditional liquid-state electrolyte (LSE), an inhomogeneous and fragile solid electrolyte interface is usually formed on the surface of the lithium metal [5,6]. The inhomogeneous interface provides nucleation sites for the growth of lithium dendrites [7,8]. Meanwhile, the fragile interfacial structure ruptures on account of the significant volume change of the lithium anode during the cycling process, exposing fresh lithium to the LSE [9]. Ultimately, the uncontrollable growth of lithium dendrites can deteriorate cycling performance and even puncture the diaphragm, causing safety hazards [10,11,12].

In addition, another key to improving the energy density of the battery is to increase the output voltage of the cathode material, while maintaining high capacity and cycling stability [13,14]. Li_3_V_2_(PO_4_)_3_ (LVP) with a monoclinic structure is an extensively studied cathode material [15,16,17]. Because of its high operating voltage, high theoretical capacity, excellent structural stability, and safety, it has become one of the candidates for a high-energy-density cathode matching lithium metal batteries [18,19,20]. Unfortunately, the LVP is prone to side reactions with the LSE under high voltage, resulting in poor cycling stability and low discharge capacity in practice [21,22]. Therewith, the application of LVP cathode material is hindered by LSE. Moreover, traditional LSE also has problems such as leakage, combustion, and toxic gas volatilization, which increase the risk of lithium metal batteries [23,24].

To solve the above challenges, one of the effective and widely investigated methods is to develop a solid-state electrolyte (SSE) [25,26,27]. To date, many SSE materials have been reported and applied to lithium batteries [28,29,30,31]. In general, SSE can be divided into two categories, namely, inorganic SSE such as sulfides and oxides [32,33], and polymeric SSE such as polyethylene-oxide-, polyvinylidene-fluoride-, and polymethyl-methacrylate-based electrolytes [34,35,36]. Among them, inorganic SSE generally has high interface resistance and low ionic conductivity at room temperature. For polymer SSE materials, although they have better contact with electrode, the electrochemical window and thermal stability need further improvement, and their compatibility with high voltage cathodes is difficult. In view of this situation, constructing hybrid SSE materials is an effective solution that combines the advantages of electrolytes with different properties [37,38,39,40].

Currently, ionic liquid has been applied in lithium batteries due to its distinct properties, including a wide electrochemical window, high thermal stability, and non-flammability, which can act as a potential electrolyte by mixing with the appropriate lithium salt [41,42,43,44]. The hybrid SSE can be obtained by adding a lithium-containing ionic liquid to an inorganic or polymer scaffold. In particular, the combination of a lithium-containing ionic liquid with a porous inorganic scaffold not only facilitates ionic conduction but can also be compatible with different cathode materials [45]. For example, Han et al. [46] designed a hybrid electrolyte with a nanowetted interface using an ordered mesoporous SiO_2_. Due to the abundant wetting interface provided by SiO_2_, it can efficiently execute ion conduction. The assembled lithium metal batteries exhibit excellent cycling performance using LiFePO_4_, LiCoO_2_, and LiNi_0_._8_Co_0_._1_Mn_0_._1_O_2_ as cathode materials. Herein, a mesoporous SiO_2_ was prepared and used as an inorganic scaffold to confine the lithium-containing ionic liquid for constructing a hybrid SSE. The ionic conductivity and electrochemical window of the as-prepared hybrid SSE were investigated. The application of the hybrid SSE in a high-operating-voltage LVP-based lithium metal battery was then studied. The excellent capacity retention demonstrates the potential of the as-prepared hybrid SSE for LVP-based lithium metal battery application.

## 2. Results and Discussion

### 2.1. Characteristic of SiO_2_ Scaffold

**Figure 1:**
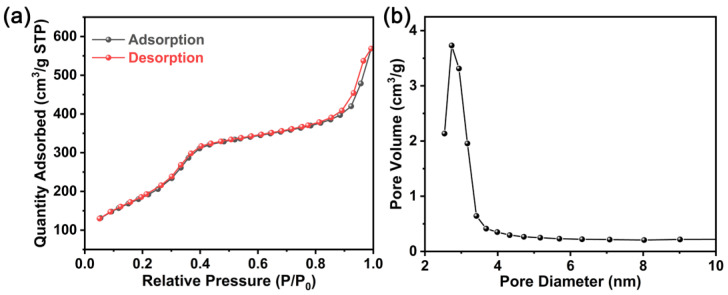
(**a**) Nitrogen absorption–desorption isotherm and (**b**) pore size distribution of the as-prepared SiO_2_.

The as-prepared SiO_2_ was characterized to ascertain its features. Firstly, the nitrogen adsorption–desorption isotherm of SiO_2_ was measured and shown in Figure 1a. As can be seen, the SiO_2_ presents a type IV isotherm, reflecting the characteristics of typical mesoporous materials. A BET-specific surface area of 912.03 m^2^ g^−1^ is received. From the pore diameter distribution curve (Figure 1b), the Barrett–Joiner–Halenda cumulative pore volume and average pore diameter are about 0.57 cm^3^ g^−1^ and 3.56 nm, respectively.

**Figure 2:**
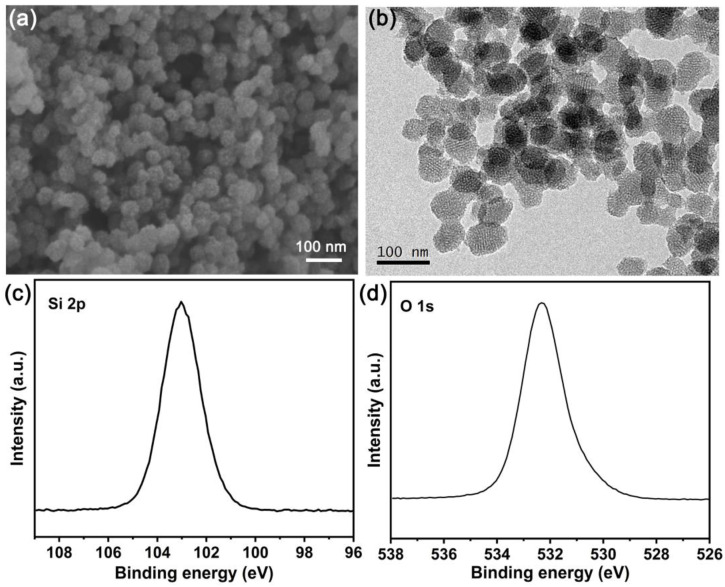
(**a**) SEM, (**b**) TEM images of SiO_2_ scaffold, and high-resolution (**c**) Si 2p and (**d**) O 1 s spectra of SiO_2_ scaffold. Si 2p and O 1 s peaks have been calibrated with C 1 s peak of adventitious carbon at binding energy of 284.8 eV.

Then, the morphology of the as-prepared SiO_2_ was investigated. As illustrated by the scanning electron microscopy (SEM) image (Figure 2a), the SiO_2_ exhibits a spherical particle with a diameter of approximately 50–60 nm. To further characterize the microstructural feature of the as-prepared SiO_2_, transmission electron microscopy (TEM) was investigated and the image is shown in Figure 2b. As can be seen, the size of the particle is about 50–60 nm, consistent with the SEM result. Moreover, the ordered mesoporous structure with a pore diameter of approximately 3–4 nm can be clearly observed, which is in agreement with the result of the nitrogen adsorption–desorption isotherm.

The X-ray photoelectron spectroscopy (XPS) data were collected to explore the elemental composition and chemical state of the as-prepared SiO_2_. From Figure 2c,d, two strong peaks attributed to Si and O are observed, which demonstrates the existence of Si and O elements in the as-prepared SiO_2_. In general, the spectrum reported by Si 2p consists of two contribution signals, which include the Si 2p_3/2_ and Si 2p_1/2_ spin orbit coupling states. However, the energy interval between the two peaks is only approximately 0.5 eV, allowing the spectrum to exhibit with a single peak [47]. As shown in Figure 2c, the Si 2p spectrum of the as-prepared SiO_2_ is detected by one peak at a binding energy of 103.0 eV, which demonstrates for the Si^4+^ species in SiO_2_ [48]. For the O 1 s photoelectron spectrum (Figure 2d), a strong peak located at a binding energy of 532.3 eV is detected in the as-prepared SiO_2_, and the signal denotes lattice oxygen.

**Figure 3:**
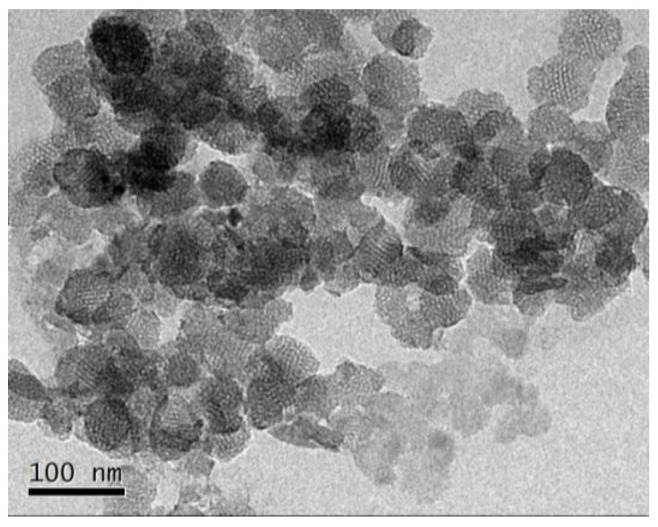
TEM images of SiO_2_ scaffold after being pressed under 10 MPa force.

In addition, the mechanical performance of the as-prepared SiO_2_ is an important parameter for the SSE. To measure the mechanical performance of the SiO_2_, it was pressed under 10 MPa force. Then, TEM was conducted and the image is shown in Figure 3. As can be seen, the porous structure of SiO_2_ is still well-maintained after high pressure, indicating that SiO_2_ has high mechanical performance. On the basis of the above results, SiO_2_ nanoparticles with many mesoporous channels are prepared successfully and can serve as a potential candidate for the scaffold in SSE.

### 2.2. Characteristic of Hybrid SSE

**Figure 4:**
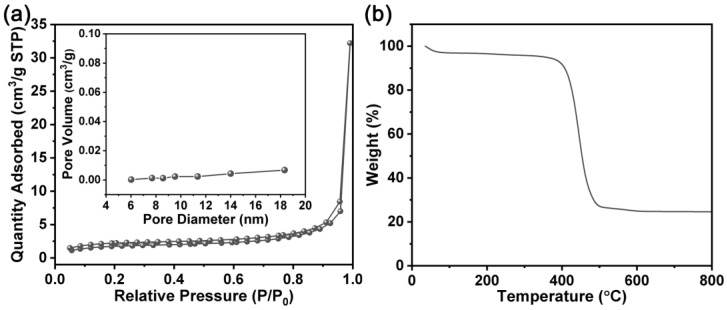
(**a**) Nitrogen absorption–desorption isotherm (inset shows the pore size distribution) and (**b**) TG curve of hybrid SSE.

To confirm whether the ionic liquid can enter the nanopores inside the SiO_2_ scaffold, the nitrogen adsorption–desorption isotherm of hybrid SSE was measured. As displayed in Figure 4a, the hybrid SSE shows an extremely low BET-specific surface area of 5.30 m^2^ g^−1^. Moreover, no obvious nanopores were reflected on the basis of the pore size distribution curve (inset in Figure 4a). The results imply that the ionic liquid can enter the nanopores inside the SiO_2_ scaffold. The thermal stability of the hybrid SSE was investigated by thermogravimetric (TG) analysis. As shown in Figure 4b, the weight of the hybrid SSE only decreases slightly in the range of room temperature to 350 °C due to the adsorbed water. When the temperature exceeds 350 °C, the hybrid SSE begins to lose its weight rapidly. The result indicates that the hybrid SSE has good thermal stability.

**Figure 5:**
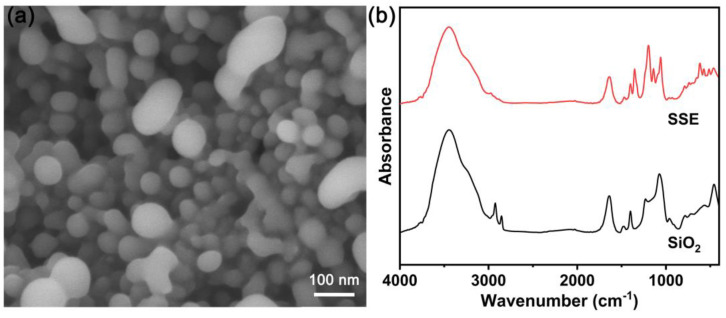
(**a**) SEM image of the hybrid SSE, and (**b**) FT-IR curves of SiO_2_ scaffold and hybrid SSE.

The morphology of the hybrid SSE was characterized by SEM. As displayed in Figure 5a, the orbicular structure of the SiO_2_ scaffold in the hybrid SSE is well-maintained. Particularly, all mesoporous channels in the hybrid SSE are filled with the ionic liquid, resulting in a smoother surface, consistent with the nitrogen adsorption–desorption isotherm result. As a contrast, the SiO_2_ scaffold has a rough surface due to the presence of mesoporous channels (Figure 2a). Due to the high loading capacity of the ionic liquid, the SiO_2_ nanoparticles are ultimately connected through the ionic liquid, which is favorable in facilitating ion conduction.

The chemical structure of the as-prepared mesoporous SiO_2_ scaffold and hybrid SSE were further characterized by Fourier-transform infrared (FT−IR) spectroscopy. Figure 5b shows the FT-IR curves of the SiO_2_ scaffold and hybrid SSE for comparison. It can be seen that SiO_2_ exhibits a strong absorption peak at about 1070 cm^−1^, corresponding to the stretching vibration of the Si−O−Si bond [49]. The absorption peaks at about 959 cm^−1^, 783 cm^−1^, and 457 cm^−1^ are all related to the vibration of the Si−O. In addition, the peak at about 1641 cm^−1^ and the wide signal between 3000–4000 cm^−1^ can be attributed to the vibration of hydroxy from the adsorbed water. After confining the ionic liquid with mesoporous SiO_2_ scaffold, the obtained hybrid SSE still retains the signal peak of SiO_2_ completely. Furthermore, the bands from the TFSI anion are observed. In detail, the peak at about 1196 cm^−1^ is indexed to the CF_3_ stretching vibration. The peaks at about 1140 cm^−1^ and 1352 cm^−1^ correspond to the vibration of the SO_2_ [50]. The results confirm that the obtained hybrid SSE inherits the structural characteristics of the SiO_2_ scaffold and ionic liquid.

**Figure 6:**
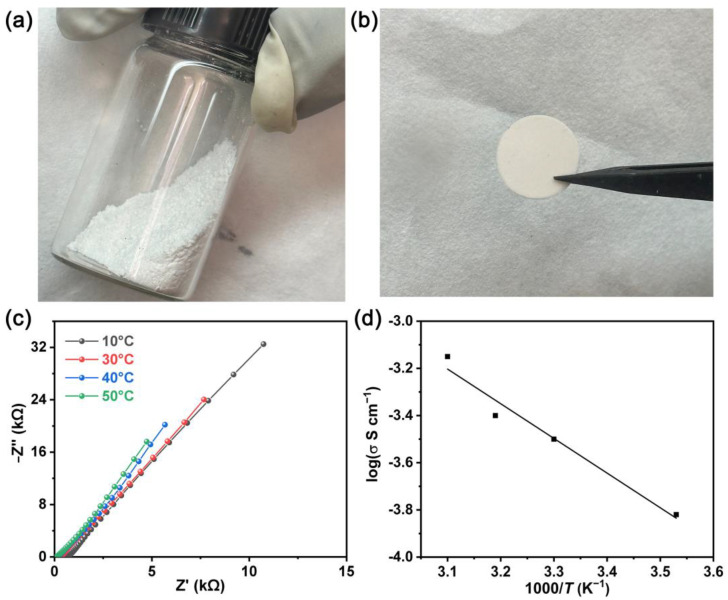
Photographs of (**a**) dry-state hybrid SSE powder and (**b**) hybrid SSE slice; (**c**) EIS plots of SS|SSE|SS symmetrical cell at different temperatures; and (**d**) the temperature dependence of ionic conductivity of the hybrid SSE. Before EIS testing, the battery is kept warm for half an hour at each temperature.

As for the hybrid SSE, ionic conductivity is one of the most important electrochemical properties. The SiO_2_ nanoparticles and lithium-containing ionic liquid were ground evenly in a glove box to obtain the hybrid SSE. From Figure 6a, the hybrid SSE presents a dry powder state. To measure the ion conductivity of the as-prepared hybrid SSE, the powder was directly pressed into a slice with a diameter of 11 mm (Figure 6b), and then assembled into a symmetrical battery with stainless steel (SS) as the electrode. Figure 6c shows the electrochemical impedance spectroscopy (EIS) plots of the SS|SSE|SS cell at different temperatures. It can be seen that all the lines show a straight tail, which presents the characteristics of the liquid electrolyte in ion conduction. The ionic conductivity of the hybrid SSE is 3.14 × 10^−4^ S cm^−1^ at 30 °C. As expected, the ion conductivity is positively associated with temperature due to the increase of the lithium ion transport rate in the electrolyte (Figure 6d). The affordable ion conductivity can be attributed to the high loading capacity of the ionic liquid and the large number of pores that provide transport channels for the lithium ion.

**Figure 7:**
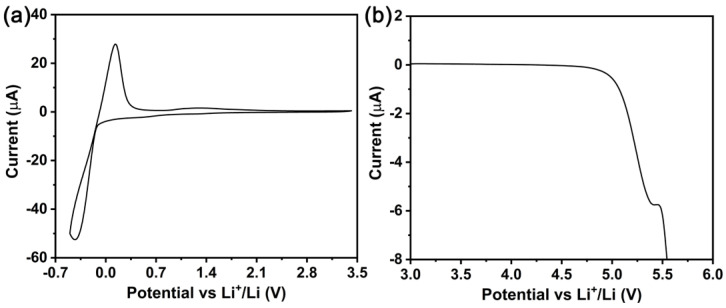
(**a**) CV and (**b**) LSV curves of Li|SSE|SS asymmetrical cell.

Obtaining an electrochemical window for the hybrid SSE is a crucial prerequisite for verifying the compatibility of the anode and cathode materials in solid-state batteries. A Li|SSE|SS asymmetrical cell was assembled and then measured by cyclic voltammetry (CV) and linear sweep voltammetry (LSV) to inspect the electrochemical potential window. As displayed in Figure 7a, the cathodic limiting potential is around 0 V. There are two obvious redox peaks in the CV curve, which agree with the previous reports [46,51]. The significant oxidation peak between 0 V and 0.5 V vs. Li^+^/Li corresponds to the stripping of the lithium ion from the SS electrode while another reduction peak indicates the plating of the lithium ion onto the SS electrode. Figure 7b shows the LSV curve of the Li|SSE|SS asymmetrical cell. As can be seen, the hybrid SSE has favorable electrochemical stability. The obvious oxidation reaction occurs only when the anodic potential exceeds 5.0 V vs. Li^+^/Li, which may be due to the chemical decomposition of the functional group in the ionic liquid, leading to the generation of a current. From this, the electrochemical potential window is approximately 5.0 V. These results imply that the hybrid SSE does not undergo reactions with the lithium metal electrode, and can be expected to be applied in a LVP-cathode-based high-voltage lithium metal battery.

### 2.3. Microstructures of LVP and Bilayer Consisting of Hybrid SSE and Cathode

**Figure 8:**
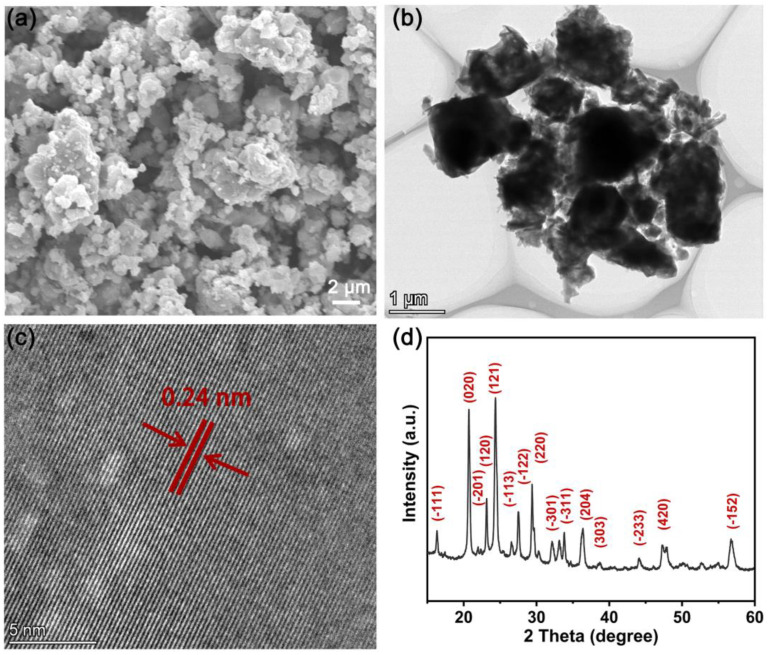
(**a**) SEM image, (**b**) TEM image, (**c**) high resolution TEM image, and (**d**) XRD pattern of LVP powder.

The LVP with a monoclinic structure has a high working voltage and theoretical specific capacity. In order to confirm the availability of the hybrid SSE, LVP was prepared by the sol–gel method, and then used as the electroactive material to assemble the battery with the hybrid SSE. Figure 8a shows the SEM image of the as-prepared LVP, which presents an irregular particle. To further expound the microstructure, a TEM test was conducted and the image is shown in Figure 8b. The obvious contrast in the image implies the surface of LVP is covered with a carbon layer. The carbon coating comes from the carbonization of citric acid, which is used as the reducing agent during the preparation of LVP.

Figure 8c exhibits the high-resolution TEM image of the LVP particle. The lattice fringes with a spacing of 0.24 nm can be clearly observed. The X-ray diffraction (XRD) pattern of the as-prepared LVP is illustrated in Figure 8d. All the diffraction peaks can be indexed to the monoclinic LVP with the *P*2_1/n_ space group, consistent with previous reports [20,52]. No diffraction peaks for carbon are observed from the XRD result, which implies the amorphous structure.

**Figure 9:**
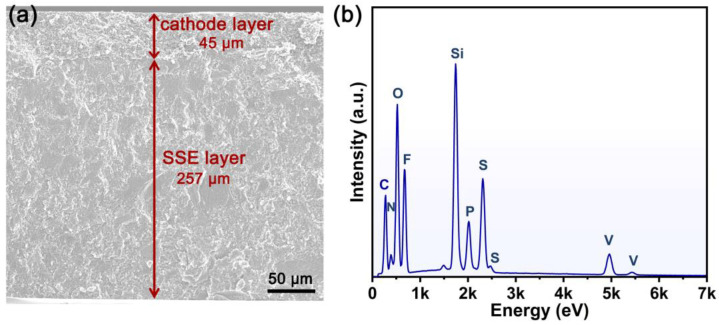
(**a**) The cross-sectional SEM image and (**b**) EDS plot of cathode and hybrid SSE bilayer structure.

Figure 9a shows the cross-sectional SEM image of the cathode and hybrid SSE bilayer structure. As can be seen, no obvious crevice is observed between the cathode layer and the hybrid SSE layer. The good contact between them is conducive to efficient interfacial ionic conduction. In addition, the elemental composition of the cathode and hybrid SSE bilayer structure was also collected (Figure 9b). The energy dispersive spectroscopy (EDS) data confirm the existence of C, O, N, F, Si, P, S, and V. Among them, the signals of N, F, and S come from the ionic liquid; while the signals of Si, P, and V reflect the presence of the SiO_2_ scaffold and LVP. The results demonstrate that a cathode and hybrid SSE bilayer structure with good contact is successfully constructed.

### 2.4. Electrochemical Performance of Lithium Metal Battery

**Figure 10:**
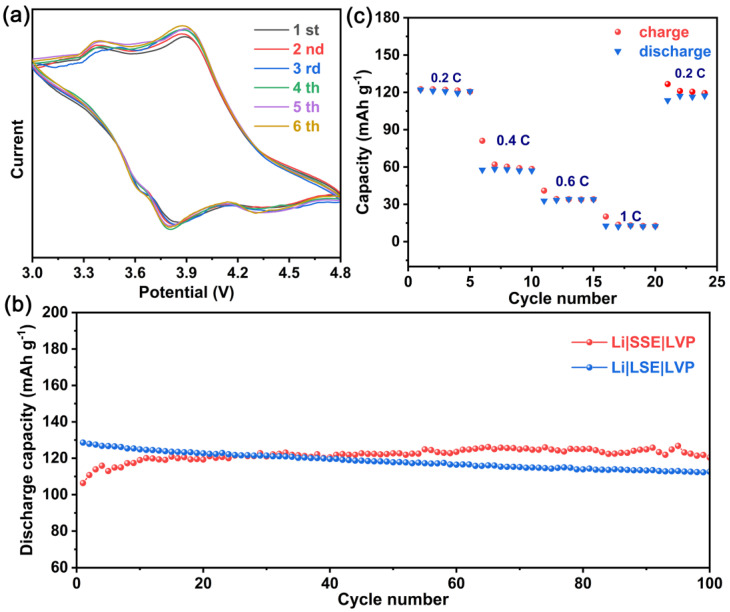
(**a**) CV curves of the Li|SSE|LVP battery at a scan rate of 0.1 mV s^−1^ during the cycling, (**b**) cycling performance of the Li|SSE|LVP and Li|LSE|LVP batteries at 0.2 C rate and room temperature, and (**c**) rate capability of Li|SSE|LVP battery at room temperature.

A Li|SSE|LVP battery was then assembled using the aforementioned bilayer structure as the electrolyte and cathode to investigate electrochemical performance. Firstly, the reversibility of the Li|SSE|LVP battery was tested by CV. As illustrated in Figure 10a, the CV curves of the Li|SSE|LVP battery are basically stable during the cycle, confirming that the employment of the hybrid SSE is beneficial to increasing the reversibility of the lithium metal battery. Moreover, the successive galvanostatic charge/discharge measurements of the battery were performed at a 0.2 C rate and room temperature. As illustrated in Figure 10b, the initial cycling of the Li|SSE|LVP battery exhibits a discharge capacity of about 106 mA h g^−1^. After 10 cycles, the discharge capacity increases to about 120 mA h g^−1^ and then remains stable. The discharge capacity is well-maintained after 100 cycles. The Li|SSE|LVP battery possesses excellent cycling performance, which verifies the hybrid SSE can effectively work with a high-voltage cathode material and shows satisfying stability.

As shown in the TEM result of LVP, a carbon coating is introduced to the LVP surface. It is well-known that carbon coating is one of the effective methods to improve the electrochemical performance [53,54]. To exclude the excellent capacity retention mainly from the surface carbon coating of the LVP and demonstrate the contribution of the hybrid SSE to stability, a Li|LSE|LVP battery was also assembled using conventional commercial LSE. Comparatively, although the Li|LSE|LVP battery delivers the higher initial discharge capacity of about 129 mA h g^−1^, its slow attenuation suffered. After 100 cycles, the discharge capacity decays to about 113 mA h g^−1^ with a capacity retention of 87.6%. The comparison of cycling performance confirms the potential of the hybrid SSE for LVP-based lithium metal battery application.

In addition, the rate capability of Li|SSE|LVP was also investigated ranging from low to high charge/discharge rates and then a return to the initial rate. Figure 10c shows the rate performance of the Li|SSE|LVP battery. As can be seen, the discharge capacity of the Li|SSE|LVP battery at a 0.2 C, 0.4 C, 0.6 C, and 1 C rate is about 120, 60, 35, and 12 mA h g^−1^, respectively. The discharge capacity of the Li|SSE|LVP battery decreases with the increase of the rate, mainly because the polarization degree increases with the increase of the current. In addition, the capacity recovers to 117 mA h g^−1^ when cycling from 1 C to 0.2 C, which is 97.5% of the initial capacity.

**Figure 11:**
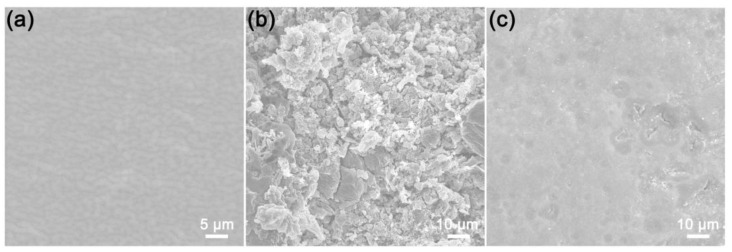
SEM images of (**a**) fresh lithium and lithium metal anode in (**b**) Li|LSE|LVP and (**c**) Li|SSE|LVP batteries after 100 cycles.

The main obstacle to the practical application of the lithium metal battery is the generation of lithium dendrites in traditional LSE and their branching into a tree-like structure, which affects electrochemical performance and even triggers safety issues. It has been reported that SSE in suppressing dendritic lithium is effective and can overcome these defects [46]. In order to compare the differences between the lithium metal anode with the application of traditional LSE and the as-prepared hybrid SSE, Li|LSE|LVP and Li|SSE|LVP batteries after charge/discharge cycle testing were disassembled in the glove box. Subsequently, the cycled Li anodes were taken out from the batteries and SEM tests to study the surface morphology were carried out immediately. Figure 11 shows the SEM images of fresh lithium and the lithium anode in Li|LSE|LVP and Li|SSE|LVP batteries after 100 cycles. As can be seen from Figure 11a, the fresh lithium presents a smooth surface. However, the lithium anode for the cycled Li|LSE|LVP battery (Figure 11b) is covered by a fragmentized lithium layer, which implies the gradual formation of dead lithium. In comparison, most of the surface of the lithium metal for the cycled Li|SSE|LVP battery (Figure 11c) remains flat and dense, and no indication of growing dendrites is observed. Although some holes have been formed, the lithium surface quality for the cycled Li|SSE|LVP battery is significantly better than that of the Li|LSE|LVP battery. The results indeed confirm the effectiveness of the as-prepared hybrid SSE in suppressing dendritic lithium and the hybrid SSE presents an excellent compatibility with the lithium anode, which is beneficial for maintaining long-term cycling performance.

## 3. Experiment

### 3.1. Preparation of SiO_2_ Scaffold

Firstly, 0.940 g of cetyltrimethylammonium bromide was dissolved in 480 mL of ultrapure water, followed by the addition of 3.5 mL of sodium hydroxide aqueous solution with a concentration of 2 M. Then, 5.5 mL of tetraethyl orthosilicate was added dropwise. After stirring at 70 °C for 3 h, the suspension was filtered to obtain a white solid and washed with ultrapure water and methanol. Subsequently, half of the dried powder was refluxed in a mixture of 60 mL of 1,4-dioxane, 17 mL of ultrapure water, and 4 mL of concentrated hydrochloric acid at 105 °C for 24 h. The SiO_2_ scaffold was obtained by filtration, washing with ultrapure water and methanol and drying at 80 °C for 48 h.

### 3.2. Preparation of SSE Powder

Firstly, a lithium-containing ionic liquid was acquired by fully dispersing 0.287 g of lithium bis(trifluoromethanesulfonyl)imide (LiTFSI) lithium salt into 2 mL of *N*-butyl-*N*-methylpyrrolidinium bis(trifluoromethanesulfonyl)imide (Pyr14TFSI). Afterwards, the above lithium-containing ionic liquid and SiO_2_ scaffold were ground thoroughly in a glove box. Then, the SSE presenting a dry powder state was obtained.

### 3.3. Preparation of LVP

0.296 g of lithium carbonate, 0.624 g of ammonium meta vanadate, and 0.920 g of ammonium dihydrogen phosphate powder were added to 100 mL of ultrapure water, and stirred quickly to form a uniform suspension. In addition, 0.806 g of citric acid was dissolved in 100 mL of ultrapure water. Subsequently, it was added to the aforementioned suspension and stirred for 24 h to form a sol. Then, a gel was formed at 80 °C, and heated at 120 °C for 12 h. After grinding the obtained powder, it was calcined at 350 °C for 4 h, and then calcined at 750 °C for 6 h in the atmosphere of nitrogen.

### 3.4. Characterization

The nitrogen absorption–desorption isotherms of SiO_2_ and hybrid SSE were recorded using a Micromeritics ASAP 2460 analyzer (Norcross, GA, USA). The morphology and chemical structure of SiO_2_ and the hybrid SSE were tested using a ZEISS Sigma 300 microscope (Oberkochen, Germany) and Thermo Scientific Nicolet iS20 infrared spectrometer (Waltham, MA, USA), respectively. The microstructural characteristic and chemical state of SiO_2_ was investigated by a FEI Tecnai F20 microscope (Hillsboro, OR, USA) and Thermo Scientific K-Alpha photoelectron spectrometer (Waltham, MA, USA), respectively. The TG experiment was performed using a TA TGA 550 analyzer (Eden Prairie, MN, USA). The morphology of the lithium metal anode, LVP, cross-sectional image of the cathode and hybrid SSE bilayer structure, and EDS data were collected by a Phenom ProX microscope equipped with an energy-dispersive X-ray detector (Amstelveen, The Netherlands). The XRD pattern of LVP was obtained using a Rigaku MiniFlex 600 X-ray diffractometer (Tokyo, Japan). The microstructure of LVP was examined using an FEI Talos F200S microscope (Hillsboro, OR, USA).

### 3.5. Fabrication of Lithium Metal Batteries

LVP, SSE powder, and conductive carbon black with a percentage of 41.7:41.7:16.6 were ground evenly in an Ar-filled glove box to obtain a composite cathode powder. After the powder with a weight of 2.4 mg was pressed into a slice on a 6 mm diameter mould, it was transferred to a 12 mm diameter mould. Subsequently, 45 mg of SSE powder was added and pressed to obtain a bilayer structure, which was used as the electrolyte and cathode. The Li|SSE|LVP battery was then assembled using a lithium metal as the anode.

As a contrast, a Li|LSE|LVP battery using commercial LSE was also assembled as follows: The LSE was 1 M LiPF_6_ dissolved in ethylene carbonate, dimethyl carbonate, and ethyl methyl carbonate with an equal volume ratio. The cathode slurry was obtained by mixing LVP, Super P, and polyvinylidene fluoride with a percentage of 80:10:10 and then coating with aluminum foil, followed by drying at 120 °C overnight under a vacuum. The coin cell was assembled with LVP as the cathode and metallic lithium as the anode. The cathode and anode were separated by a Ceglard 2325 membrane.

### 3.6. Electrochemical Measurements

The CV, LSV, and EIS were investigated using a Chenhua CHI 660E electrochemical workstation (China). Among them, the CV and LSV of the Li|SSE|SS asymmetrical cell were performed at a scan rate of 0.2 mV s^−1^. The EIS of the SS|SSE|SS symmetrical cell was measured at a frequency range of 10 Hz–1000 kHz with AC amplitude of 5 mV. The CV of the Li|SSE|LVP full cell was performed at a scan rate of 0.1 mV s^−1^. The galvanostatic charge/discharge experiments were conducted on a Land CT3002A cell test system (China) in the voltage window from 3.0 V to 4.8 V.

## 4. Conclusions

In summary, a dry-state powder was successfully obtained by the physical blending of a lithium-containing ionic liquid and mesoporous SiO_2_ scaffold, and was used as a hybrid SSE. The as-prepared hybrid SSE exhibits a high ionic conductivity and wide electrochemical window. In addition, the hybrid SSE also presents good compatibility with the LVP cathode material and lithium metal. Moreover, the assembled Li|SSE|LVP lithium metal battery delivers excellent cycling performance even when operating at a high voltage of 4.8 V. After 100 cycles, a discharge capacity retention of about 100% is achieved at a 0.2 C rate and room temperature. The excellent cycling performance can be attributed to the effectiveness of the hybrid SSE in suppressing lithium anode deterioration. The work can be a simple strategy to construct an effective hybrid SSE with desirable properties for the application in LVP-based lithium metal batteries.

## Data Availability

The data presented in this study are available upon request from the corresponding author.

## References

[1] Liu B., Zhang J.G., Xu W. (2018). Advancing lithium metal batteries. Joule.

[2] Cheng X.B., Liu H., Yuan H., Peng H.J., Tang C., Huang J.Q., Zhang Q. (2021). A perspective on sustainable energy materials for lithium batteries. SusMat.

[3] Rodriguez J.R., Kim P.J., Kim K., Qi Z., Wang H., Pol V.G. (2021). Engineered heat dissipation and current distribution boron nitride-graphene layer coated on polypropylene separator for high performance lithium metal battery. J. Colloid Interf. Sci..

[4] Zhao W., Zhang K., Wu F., Wang X., Guo R., Zhang K., Yuan Y., Bai Y., Wu C. (2023). Moisture-assistant chlorinated separator with dual-protective interface for ultralong-life and high-rate lithium metal batteries. Chem. Eng. J..

[5] Meng J., Chu F., Hu J., Li C. (2019). Liquid polydimethylsiloxane grafting to enable dendrite-free Li plating for highly reversible Li-metal batteries. Adv. Funct. Mater..

[6] Park H., Jeon Y., Chung W.J., Bae Y., Kim J., Baek H., Park J. (2022). Early stage Li plating by liquid phase and cryogenic transmission electron microscopy. ACS Energy Lett..

[7] Guan X., Wang A., Liu S., Li G., Liang F., Yang Y.W., Liu X., Luo J. (2018). Controlling nucleation in lithium metal anodes. Small.

[8] Niu S., Zhang S.W., Li D., Wang X., Chen X., Shi R., Shen N., Jin M., Zhang X., Lian Q. (2022). Sandwiched Li plating between Lithiophilic-Lithiophobic gradient Silver@ Fullerene interphase layer for ultrastable lithium metal anodes. Chem. Eng. J..

[9] Lang S.Y., Shen Z.Z., Hu X.C., Shi Y., Guo Y.G., Jia F.F., Wang F.Y., Wen R., Wan L.J. (2020). Tunable structure and dynamics of solid electrolyte interphase at lithium metal anode. Nano Energy.

[10] Ni S., Tan S., An Q., Mai L. (2020). Three dimensional porous frameworks for lithium dendrite suppression. J. Energy Chem..

[11] Zhang H., Huang L., Xu H., Zhang X., Chen Z., Gao C., Lu C., Liu Z., Jiang M., Cui G. (2022). A polymer electrolyte with a thermally induced interfacial ion-blocking function enables safety-enhanced lithium metal batteries. eScience.

[12] Zhang W., Long J., Wang H., Lan J., Yu Y., Yang X. (2022). Novel in situ growth of ZIF-8 in porous epoxy matrix for mechanically robust composite electrolyte of high-performance, long-life lithium metal batteries. Molecules.

[13] Li H., Xu M., Zhang Z., Lai Y., Ma J. (2020). Engineering of polyanion type cathode materials for sodium-ion batteries: Toward higher energy/power density. Adv. Funct. Mater..

[14] Chang Z., Qiao Y., Yang H., Deng H., Zhu X., He P., Zhou H. (2020). Beyond the concentrated electrolyte: Further depleting solvent molecules within a Li^+^ solvation sheath to stabilize high-energy-density lithium metal batteries. Energy Environ. Sci..

[15] Rui X., Yan Q., Skyllas-Kazacos M., Lim T.M. (2014). Li_3_V_2_(PO_4_)_3_ cathode materials for lithium-ion batteries: A review. J. Power Sources.

[16] Liang M., Li L., Cui X., Qi S., Wang L., Dong H., Chen X., Wang Y., Chen S., Wang G. (2022). Ru- and Cl-codoped Li_3_V_2_(PO_4_)_3_ with enhanced performance for lithium-ion batteries in a wide temperature range. Small.

[17] Ruan T., Lu S., Lu J., Niu J., Li R. (2023). Unraveling the intercalation chemistry of multi-electron reaction for polyanionic cathode Li_3_V_2_(PO_4_)_3_. Energy Storage Mater..

[18] Su A., Guo P., Li J., Kan D., Pang Q., Li T., Sun J., Chen G., Wei Y. (2020). An organic-inorganic semi-interpenetrating network ionogel electrolyte for high-voltage lithium metal batteries. J. Mater. Chem. A.

[19] Huang Z., Luo P., Zheng H., Lyu Z. (2022). Aluminum-doping effects on three-dimensional Li_3_V_2_(PO_4_)_3_@C/CNTs microspheres for electrochemical energy storage. Ceram. Int..

[20] Ding M., Cheng C., Wei Q., Hu Y., Yan Y., Dai K., Mao J., Guo J., Zhang L., Mai L. (2021). Carbon decorated Li_3_V_2_(PO_4_)_3_ for high-rate lithium-ion batteries: Electrochemical performance and charge compensation mechanism. J. Energy Chem..

[21] Han H., Qiu F., Liu Z., Han X.E. (2015). ZrO_2_-coated Li_3_V_2_(PO_4_)_3_/C nanocomposite: A high-voltage cathode for rechargeable lithium-ion batteries with remarkable cycling performance. Ceram. Int..

[22] Rajagopalan R., Zhang L., Dou S.X., Liu H. (2016). Lyophilized 3D lithium vanadium phosphate/reduced graphene oxide electrodes for super stable lithium ion batteries. Adv. Energy Mater..

[23] Zhang Q.K., Zhang X.Q., Yuan H., Huang J.Q. (2021). Thermally stable and nonflammable electrolytes for lithium metal batteries: Progress and perspectives. Small Sci..

[24] Zhang Q.K., Zhang X.Q., Hou L.P., Sun S.Y., Zhan Y.X., Liang J.L., Zhang F.S., Feng X.N., Li B.Q., Huang J.Q. (2022). Regulating solvation structure in nonflammable amide-based electrolytes for long-cycling and safe lithium metal batteries. Adv. Energy Mater..

[25] Xia S., Wu X., Zhang Z., Cui Y., Liu W. (2019). Practical challenges and future perspectives of all-solid-state lithium-metal batteries. Chem.

[26] Chen X., Guan Z., Chu F., Xue Z., Wu F., Yu Y. (2022). Air-stable inorganic solid-state electrolytes for high energy density lithium batteries: Challenges, strategies, and prospects. InfoMat.

[27] Wei T., Wang Z.M., Zhang Q., Zhou Y., Sun C., Wang M., Liu Y., Wang S., Yu Z., Qiu X. (2022). Metal-organic frameworks-based solid-state electrolytes for all solid-state lithium metal batteries: A review. CrystEngComm.

[28] Zhao W., Yi J., He P., Zhou H. (2019). Solid-state electrolytes for lithium-ion batteries: Fundamentals, challenges and perspectives. Electrochem. Energ. Rev..

[29] Huang W.H., Li X.M., Yang X.F., Zhang X.X., Wang H.H., Wang H. (2021). The recent progress and perspectives on metal-and covalent-organic framework based solid-state electrolytes for lithium-ion batteries. Mater. Chem. Front..

[30] Xu L., Li J., Deng W., Shuai H., Li S., Xu Z., Li J., Hou H., Peng H., Ji X. (2021). Garnet solid electrolyte for advanced all-solid-state Li batteries. Adv. Energy Mater..

[31] Yang S.Y., Shadike Z., Wang W.W., Yue X.Y., Xia H.Y., Bak S.M., Du Y.H., Li H., Fu Z.W. (2022). An ultrathin solid-state electrolyte film coated on LiNi_0.8_Co_0.1_Mn_0.1_O_2_ electrode surface for enhanced performance of lithium-ion batteries. Energy Storage Mater..

[32] Zhang Q., Cao D., Ma Y., Natan A., Aurora P., Zhu H. (2019). Sulfide-based solid-state electrolytes: Synthesis, stability, and potential for all-solid-state batteries. Adv. Mater..

[33] Su H., Jiang Z., Liu Y., Li J., Gu C., Wang X., Xia X., Tu J. (2022). Recent progress of sulfide electrolytes for all-solid-state lithium batteries. Energy Mater..

[34] Zhu M., Wu J., Wang Y., Song M., Long L., Siyal S.H., Yang X., Sui G. (2019). Recent advances in gel polymer electrolyte for high-performance lithium batteries. J. Energy Chem..

[35] Bao W., Hu Z., Wang Y., Jiang J., Huo S., Fan W., Chen W., Jing X., Long X., Zhang Y. (2022). Poly (ionic liquid)-functionalized graphene oxide towards ambient temperature operation of all-solid-state PEO-based polymer electrolyte lithium metal batteries. Chem. Eng. J..

[36] Wang Y., Huang K., Zhang P., Li H., Mi H. (2022). PVDF-HFP based polymer electrolytes with high Li^+^ transference number enhancing the cycling performance and rate capability of lithium metal batteries. Appl. Surf. Sci..

[37] Lv F., Wang Z., Shi L., Zhu J., Edström K., Mindemark J., Yuan S. (2019). Challenges and development of composite solid-state electrolytes for high-performance lithium ion batteries. J. Power Sources.

[38] Zhang T., He W., Zhang W., Wang T., Li P., Sun Z., Yu X. (2020). Designing composite solid-state electrolytes for high performance lithium ion or lithium metal batteries. Chem. Sci..

[39] Zheng Y., Yao Y., Ou J., Li M., Luo D., Dou H., Li Z., Amine K., Yu A., Chen Z. (2020). A review of composite solid-state electrolytes for lithium batteries: Fundamentals, key materials and advanced structures. Chem. Soc. Rev..

[40] Wang B., Wang G., He P., Fan L.Z. (2022). Rational design of ultrathin composite solid-state electrolyte for high-performance lithium metal batteries. J. Membrane Sci..

[41] Lu Y., Das S.K., Moganty S.S., Archer L.A. (2012). Ionic liquid-nanoparticle hybrid electrolytes and their application in secondary lithium-metal batteries. Adv. Mater..

[42] Yang G., Song Y., Wang Q., Zhang L., Deng L. (2020). Review of ionic liquids containing, polymer/inorganic hybrid electrolytes for lithium metal batteries. Mater. Design.

[43] Yu D., Ma Z., Liu Z., Jiang X., Younus H.A., Wang X., Zhang S. (2023). Optimizing interfacial wetting by ionic liquid for high performance solid-state lithium metal batteries operated at ambient temperature. Chem. Eng. J..

[44] Tang X., Lv S., Jiang K., Zhou G., Liu X. (2022). Recent development of ionic liquid-based electrolytes in lithium-ion batteries. J. Power Sources.

[45] Li J., Li F., Zhang L., Zhang H., Lassi U., Ji X. (2021). Recent applications of ionic liquids in quasi-solid-state lithium metal batteries. Green Chem. Eng..

[46] Han L., Wang Z., Kong D., Yang L., Yang K., Wang Z., Pan F. (2018). An ordered mesoporous silica framework based electrolyte with nanowetted interfaces for solid-state lithium batteries. J. Mater. Chem. A.

[47] Cañón J., Teplyakov A.V. (2021). XPS characterization of cobalt impregnated SiO_2_ and γ-Al_2_O_3_. Surf. Interface Anal..

[48] Xiang Z., He Q., Wang Y., Yin X., Xu B. (2022). Preparation and electromagnetic wave absorption properties of SiC/SiO_2_ nanocomposites with different special structures. Appl. Surf. Sci..

[49] Katcharava Z., Marinow A., Bhandary R., Binder W.H. (2022). 3D Printable Composite Polymer Electrolytes: Influence of SiO_2_ Nanoparticles on 3D-Printability. Nanomaterials.

[50] Kim K., Kuhn L., Alabugin I.V., Hallinan Jr D.T. (2020). Lithium salt dissociation in diblock copolymer electrolyte using fourier transform infrared spectroscopy. Front. Energy Res..

[51] Choudhury S., Mangal R., Agrawal A., Archer L.A. (2015). A highly reversible room-temperature lithium metal battery based on crosslinked hairy nanoparticles. Nat. Commun..

[52] Liu Q., Yang F., Wang S., Feng L., Zhang W., Wei H. (2013). A simple diethylene glycol-assisted synthesis and high rate performance of Li_3_V_2_(PO_4_)_3_/C composites as cathode material for Li-ion batteries. Electrochim. Acta.

[53] Liao Y., Li C., Lou X., Hu X., Ning Y., Yuan F., Hu B. (2018). Carbon-coated Li_3_V_2_(PO_4_)_3_ derived from metal-organic framework as cathode for lithium-ion batteries with high stability. Electrochim. Acta.

[54] Mohanty D., Lu Z.L., Hung I.M. (2023). Effect of carbon coating on electrochemical properties of Li_3_V_2_(PO_4_)_3_ cathode synthesized by citric-acid gel method for lithium-ion batteries. J. Appl. Electrochem..

